# 

**Published:** 1905-04-15

**Authors:** 


### CONTENTS.

Event and Comment - - - - - - - - - - - 163
How Do You Fill Pulp Canals? - By A. W. Harlan, M.D., D.D.S. 171
The Value of Chemistry in Dentistry, - - By George M. Heath 178
Pressure Anesthesia, - - - - By J. V. Conzett, D.D.S. 186
Difficulties of Pressure Anesthesia, - - By J. B. Pherrin, D.D.S. 190
Liberation or Extraction of Impacted Teeth,
By M. H. Cryer, M.D., D.D.S. 193
Treatment of Compound Fracture of the Jaws, By Dr. T. L. Gilmer 195
Degeneracy, - - - - - By E. S. Talbot, M.D., D.D.S. 196
Announcements - - - - - - - - - - - 198
Obituary - - - - - - - - - - - 200
Indents - - - - - - - - - - - 202

